# Stem/Progenitor Cells in Cardiopulmonary Health, Disease, and Treatment

**DOI:** 10.1155/2019/9861403

**Published:** 2019-01-06

**Authors:** Fatemeh Sharifpanah, Hossein A. Ghofrani, Suk Ying Tsang, Heinrich Sauer

**Affiliations:** ^1^Department of Physiology, Faculty of Medicine, Justus Liebig University, Giessen, Germany; ^2^Department of Internal Medicine, Justus Liebig University Giessen, Universities of Giessen and Marburg Lung Center (UGMLC), German Center for Lung Research (DZL), Giessen, Germany; ^3^Department of Medicine, Imperial College London, London, UK; ^4^School of Life Sciences, The Chinese University of Hong Kong, Hong Kong; ^5^Key Laboratory for Regenerative Medicine, Ministry of Education, The Chinese University of Hong Kong, Hong Kong; ^6^State Key Laboratory of Agrobiotechnology, The Chinese University of Hong Kong, Hong Kong; ^7^Centre for Novel Biomaterials, The Chinese University of Hong Kong, Hong Kong

The cardiopulmonary system comprises various organs, structures, and substances from both the heart and lung systems. Since the cardiopulmonary system interacts intimately with every other system in the body and our health is closely related to the function of the cardiopulmonary system, its function and maintenance were always the center of attention by scientists and clinicians. Despite plentiful research in this area, cardiopulmonary diseases still remain widely prevalent and are known as a significant and devastating cause of morbidity and mortality on the globe. The most common cardiopulmonary diseases are hypertension, chronic obstructive pulmonary disease, coronary heart disease, and rheumatic heart fever. Generally, cardiopulmonary disorders have a poor prognosis and current treatments only offer a modest improvement of symptoms without repairing the damaged tissues in the heart or lung system. But recent progress in the field of stem cell science, regenerative medicine, and tissue engineering offered a new perspective in the treatment of cardiopulmonary disorders.

This great achievement provided tremendous potential to develop disease- and patient-specific induced pluripotent stem (iPS) cells as well as organoid cultures for gathering detailed insight into the pathomechanisms of various cardiopulmonary disorders which is important in drug discovery and treatment of patients. Furthermore, the revolutionized progress in the stem cell field has opened a great opportunity for novel personalized regenerative therapeutic approaches to treat cardiopulmonary disorders. There is abundant and even growing number of evidence in stem/progenitor cell therapy of various cardiopulmonary disorders of which some generated controversial information. To summarize and incorporate these scattered data and develop a more comprehensive understanding on the impact of stem/progenitor cells in the cardiopulmonary system, the *Stem Cells International* journal sets out to publish a special issue focused on “Stem/Progenitor Cells in Cardiopulmonary Health, Disease, and Treatment” which covers research from diverse disciplines related to cardiopulmonary health. This special issue sums up recent findings concerning the essential role of stem/progenitor cells in the cardiac as well as pulmonary system. It includes selected reviews and original articles discussing the role and properties of stem/progenitor cells not only from the perspective of basic science but also from the perspective of therapeutic strategies.

As known, the human adult heart lacks a robust endogenous repair mechanism and is not able to regenerate and repair itself after injuries. Therefore, developing and establishing approaches to regenerate and repair the injured heart are a top priority in treating heart failure. One of the most promising therapeutic strategies for cardiac injury treatment is cell therapy. But before using cell therapy for treating patients, it is necessary to be able to efficiently generate a sufficient number of functional cardiomyocytes which are capable to functionally and safely integrate into the injured area of the heart. The recently developed “direct cardiac reprogramming” technique is a promising approach for generating functional cardiomyocytes. J. L. Engel and R. Ardehali on their review article entitled “Direct Cardiac Reprogramming: Progress and Promise” have comprehensively reviewed the key transcription factors and cardiogenic genes as well as various biological molecules (e.g., epigenetic modifiers, noncoding RNAs, and small molecules) which improve the efficiency of cardiac reprogramming to develop safer reprogramming approaches for future clinical applications. In this context, Y. Zhou et al. have examined the function of various epigenetic factors involved in chromatin remodeling and RNA splicing to identify the inhibitory or facilitating effects of these factors in direct cardiac reprogramming. As they have reported on their interesting research article entitled “A Loss of Function Screen of Epigenetic Modifiers and Splicing Factors during Early Stage of Cardiac Reprogramming,” the splicing factors Sf3a1 and Sf3b1 are essential regulators for direct reprogramming, while the splicing factor Zrsr2 as well as the epigenetic modulators Bcor and Stag2 functions as inhibitory barriers for direct cardiac reprogramming. They have finally highlighted the impact of epigenetic regulation and the RNA splicing process during cell fate conversion. As it is also discussed by J. L. Engel and R. Ardehali, small molecules can be used to improve the efficiency of cardiac regeneration. Various small molecules are intensively investigated by different groups for more profound understanding of the cardiac regeneration process which can be potentially used for the development of new therapeutic strategies for heart failure treatment. In this regard, E. H. Ali et al. have investigated the translational potential of the small molecule silibinin in cardiomyogenesis of embryonic stem cells. The small molecule silibinin is a natural compound isolated from milk thistle seed extracts and is traditionally used as a hepatoprotectant. In research article “The Milk Thistle (*Silybum marianum*) Compound Silibinin Inhibits Cardiomyogenesis of Embryonic Stem Cells by Interfering with Angiotensin II Signaling” authored by E. H. Ali et al., it has been demonstrated that small molecule silibinin as an inhibitor of the angiotensin II type 1 (AT1) receptor inhibits the cardiomyogenesis process of embryonic stem cells by interfering with angiotensin II signaling downstream of the AT1 receptor.

Amongst multiple therapeutic approaches and various pharmacological agents which have shown an improvement in patient care and decrease of mortality rate, stem/progenitor cell-based therapeutic approaches present the most promising approach to treat the cardiopulmonary disorders in the future. S. Bardelli and M. Moccetti on their article entitled “Stem and Progenitor Cells in Human Cardiopulmonary Development and Regeneration” have briefly reviewed the contribution of stem/progenitor cells in the development of the cardiopulmonary system as well as the cellular plasticity potential of stem/progenitor cells in the regeneration of the injured heart and lung organs and their current therapeutic applications in the treatment of cardiopulmonary diseases. In this regard, N. Witman and M. Sahara in the review article entitled “Cardiac Progenitor Cells in Basic Biology and Regenerative Medicine” have comprehensively discussed the biology of embryonic and adult cardiac progenitor cells and their regenerative capabilities as well as their current clinical applications in cardiac repair. Cardiac progenitor cells are a heterogeneous cell population distributed throughout the heart which are quiescent under physiological conditions and become activated after injury and may differentiate into new myocytes and vascular cells [1]. J. L. Engel and R. Ardehali review a novel technique which is generating expandable cardiac progenitor cells by direct reprogramming in culture for transplantation in their abovementioned review article.

Mesenchymal stem cells (MSCs), the major stem cells for regenerative cell-based therapy, have been characterized by Dr. Alexander Friedenstein over 40 years ago and are widely used for treating a variety of diseases. Along with the immunomodulatory and differentiation potential of MSCs, it has been shown that they express various essential cytokines which stimulate local tissue repair [2]. The review made by J. Gronbach et al. entitled “The Potentials and Caveats of Mesenchymal Stromal Cell-Based Therapies in the Preterm Infant” has essentially described the role of stem cells in lung development as well as pulmonary diseases. They have further discussed the potential and challenges of mesenchymal stem cell-based therapies in preterm infants and explained the positive effects of exogenous MSC therapy in the diseased lung with the special focus on the bronchopulmonary dysplasia, a chronic lung disease of preterm infants. Although MSC-based therapies are a highly promising approach for treating patients, the authors explained that still a lot of research is needed to bring optimized MSC products into upcoming clinical trials and their outcomes have to be critically evaluated before a broad introduction of MSC-based therapies into the clinics can be achieved. One of the most important points for optimizing MSC products is maintenance and expansion of MSCs in culture before transplantation. With a well optimized protocol, it will be possible to generate a sufficient cell number with the best quality needed for transplantation. Despite regulatory issues about using animal-derived cell culture supplements in clinic, most clinical trials of MSC-based cell therapies are still dependent on fetal bovine serum for cell expansion before transplantation. In order to solve this problem, T. Z. Nazari-Shafti et al. have investigated the effect of human serum from heart failure patients and also healthy donors on cord blood MSCs during regular short-term cultivation as well as stimulated acute and chronic stress conditions and compared their results with the findings from cells treated with fetal bovine serum in the same conditions on their research article entitled “Mesenchymal Stromal Cells Cultured in Serum from Heart Failure Patients Are More Resistant to Simulated Chronic and Acute Stress.” They have demonstrated that autologous human serum will be a valid alternative to fetal bovine serum in cell-based therapies addressing severe heart disease.

Cell-based therapies using MSCs are widely used for treating various pathological conditions. One of the recent usages of MSCs is in sepsis. G. M. Galstian et al. have recently shown that treatment of neutropenic patients with MSCs during the first hours of septic shock might improve their short-term survival, but cannot prevent decease due to long-term sepsis-related organ dysfunctions [3]. In the present special issue, J. Horák et al. have explained in detail the properties of MSCs and their mechanisms of action in physiological conditions and in sepsis on their article entitled “Mesenchymal Stem Cells in Sepsis and Associated Organ Dysfunction: A Promising Future or Blind Alley?” They have further discussed the beneficial effect of MSCs on the cardiovascular system as well in sepsis-associated organ dysfunction with a special focus on acute kidney injury which is the most frequent complication of sepsis. Based on recent literature, they have suggested that MSCs with their anti-inflammatory actions are able to reverse detrimental effects of endotoxemia in the heart. They have finally recommended continued evaluation of the safety of MSCs in treatment of sepsis for further translational research in the form of multicenter projects in several world renowned laboratories for additional justification and assurance.

Another useful stem/progenitor cell type which is also commonly used in cell therapy approaches is bone marrow-derived CD34-positive cells. CD34-positive cells are a well-characterized population of stem cells which are traditionally used to reconstitute hematopoietic cells after radiation or chemotherapy. The capability of CD34-positive cells in the induction of therapeutic angiogenesis is reported in a variety of animal models and includes pathological conditions like myocardial, peripheral, and cerebral ischemia as well as acute lung injury [4, 5]. Furthermore, different studies have shown promising results from treatment with CD34-positive cells in various clinical trials on patients with ischemic diseases. Despite increasing numbers of successful clinical trials which confirm the clinical significance of autologous CD34-positive cell therapy and introduce them as potent contributors in vascular, cardiovascular, and pulmonary repair, the selection of optimal time points, cell dosage, and route of administration still remains unclear. An optimized and common evaluation technique on therapeutic efficacy of cell therapy can help to solve these problems. In an interesting in vivo study, T.-H. Huang et al. on their article “Correlation between Therapeutic Efficacy of CD34^+^ Cell Treatment and Directed *In Vivo* Angiogenesis in Patients with End-Stage Diffuse Coronary Artery Disease” have investigated the therapeutic efficacy of CD34-positive cell treatment in patients with end-stage diffuse coronary artery disease as reflected in the rate of angiogenesis/neovascularization pre- and posttreatment with autologous CD34-positive cells. This method is known as “angiographic grading”. T.-H. Huang et al. have carefully compared the results of the angiogenic grading method with findings of the “directed in vivo angiogenesis assay (DIVAA),” which is a known quantitative assessment technique for angiogenic responses. They have finally suggested the DIVAA method as a better and reliable technique than angiographic grading for assessing coronary vascularization after CD34-positive cell treatment. DIVAA has a potential to become routine for assessment of angiogenic responses in the clinical setting.

Exosomes are cell-derived extracellular vesicles which can be found in perhaps all eukaryotic fluids and provide a means of intercellular communication. They correspond to intraluminal vesicles (ILVs) which are formed within intracellular multivesicular bodies (MVBs). ILVs are generated by invagination of the endosomal membrane via a dependent and/or independent manner to the endosomal sorting complexes required for transport. ILVs and exosomes as well will release from the cell into the extracellular space upon fusion of MVBs with plasma membrane. The specific contents of exosomes (e.g., RNAs, lipids, and proteins) which only originate from the cell cytosol or endosomal compartments appear to be as diverse as cell types and are highly dependent to the state of the cell and its changes. Exosomes can be potentially used as biomarkers for prognosis of different diseases and also for therapeutic purposes [6, 7]. J. A. Maring et al. contributed with a review entitled “Myocardial Regeneration via Progenitor Cell-Derived Exosomes” where they have broadly described the nature of exosomes in their review article and also explained the current state of progenitor cell-derived exosomes in experimental therapy of heart diseases and their translational potential in myocardial regeneration.

In agreement with the results of increasing numbers of clinical trials and various experimental reports, adult stem cell-based therapy is a promising novel approach for the treatment of various diseases. Biomaterial scaffolds can support the transplantation of stem cell by providing not only a physical support for transplanted cells but also chemical and physiological cues for the cells to facilitate tissue regeneration. The recent development of tissue and biomaterial engineering during last years resulted in a revolution in the field of regenerative medicine and gives a promising future in the field of organ regeneration and transplantation which can secure a better life quality for the patients. Despite various valuable studies in the field of tissue engineering, there is still a long way to find the best and desired biomaterial scaffolds which should be seeded with sufficient numbers of suitable stem cell types to assemble functional engineered constructs that preserve, maintain, and improve damaged tissue or whole organs. Within this special issue, A. O. J. Fakoya et al. published the review article entitled “Current Trends in Biomaterial Utilization for Cardiopulmonary System Regeneration” in which they have broadly discussed the current natural and synthetic biomaterial scaffolds which are utilized to regenerate cardiac and pulmonary organs. They have further highlighted advantages and disadvantages of each utilized biomaterial scaffold for cardiopulmonary system regeneration. This comprehensive review can be an insight to find out the best choice of biomaterial scaffolds to generate functional heart and lung tissue batched for transplantation.

In summary, this special issue offers a compact overview of the major findings concerning the impact of stem/progenitor cells in health, disease, and treatment of cardiopulmonary diseases. The recent developments should lead to new scientific insights into the function of stem/progenitor cells in the context of potential therapeutic applications and next generation stem cell therapies for cardiopulmonary disorders. Combined and continued endeavor between basic, translational, and clinical research will hopefully generate an innovative and efficient means for the treatment or even cure of devastating cardiopulmonary diseases.
